# The impact of parental risk factors on the risk of stroke in type 1 diabetes

**DOI:** 10.1007/s00592-021-01694-x

**Published:** 2021-03-15

**Authors:** Anni Ylinen, Stefanie Hägg-Holmberg, Marika I. Eriksson, Carol Forsblom, Valma Harjutsalo, Jukka Putaala, Per-Henrik Groop, Lena M. Thorn, on behalf of the FinnDiane Study Group

**Affiliations:** 1 Folkhälsan Institute of Genetics, Folkhälsan Research Center, Biomedicum Helsinki, Helsinki, Finland; 2 Department of Nephrology, University of Helsinki and Helsinki University Hospital, Helsinki, Finland; 3 Research Program for Clinical and Molecular Metabolism, Faculty of Medicine, University of Helsinki, Helsinki, Finland; 4 National Institute for Health and Welfare, Helsinki, Finland; 5 Helsinki University Hospital and University of Helsinki, Neurology, Helsinki, Finland; 6 Department of Diabetes, Central Clinical School, Monash University, Melbourne, VIC Australia; 7 Department of General Practice and Primary Health Care, University of Helsinki and Helsinki University Hospital, Helsinki, Finland

**Keywords:** Parental risk factors, Cardiovascular complications, Stroke, Hemorrhagic stroke, Ischemic stroke, Type 1 diabetes

## Abstract

**Background:**

Individuals with type 1 diabetes have a markedly increased risk of stroke. In the general population, genetic predisposition has been linked to increased risk of stroke, but this has not been assessed in type 1 diabetes. Our aim was, therefore, to study how parental risk factors affect the risk of stroke in individuals with type 1 diabetes.

**Methods:**

This study represents an observational follow-up of 4011 individuals from the Finnish Diabetic Nephropathy Study, mean age at baseline 37.6 ± 11.9 years. All strokes during follow-up were verified from medical records or death certificates. The strokes were classified as either ischemic or hemorrhagic. All individuals filled out questionnaires concerning their parents’ medical history of hypertension, diabetes, stroke, and/or myocardial infarction.

**Results:**

During a median follow-up of 12.4 (10.9–14.2) years, 188 individuals (4.6%) were diagnosed with their first ever stroke; 134 were ischemic and 54 hemorrhagic. In Cox regression analysis, a history of maternal stroke increased the risk of hemorrhagic stroke, hazard ratio 2.86 (95% confidence interval 1.27–6.44, *p* = 0.011) after adjustment for sex, age, BMI, retinal photocoagulation, and diabetic kidney disease. There was, however, no association between maternal stroke and ischemic stroke. No other associations between parental risk factors and ischemic or hemorrhagic stroke were observed.

**Conclusion:**

A history of maternal stroke increases the risk of hemorrhagic stroke in individuals with type 1 diabetes. Other parental risk factors seem to have limited impact on the risk of stroke.

**Supplementary Information:**

The online version contains supplementary material available at (10.1007/s00592-021-01694-x).

## Background

Diabetes is a significant risk factor for stroke, especially in young people [1]. The incidence of stroke in individuals with type 1 diabetes is 310–475 per 100,000 person-years [2–4], which is about fivefold higher than in the general population [5]. Stroke is a cerebrovascular disease commonly divided into two subgroups, ischemic and hemorrhagic. Hemorrhagic stroke is less common than ischemic stroke but associated with worse prognosis and higher mortality rate [6]. Risk factors for stroke, such as age, hypertension, and smoking are similar in the general population [7, 8] as in type 2 [9] and type 1 diabetes [10]. One of the traditional risk factors for stroke, atrial fibrillation, does not relate to increased risk of stroke in type 1 diabetes, at least in younger individuals [10]. In type 1 diabetes, however, also diabetes-related factors increase the risk of stroke, and the risk factor profiles for ischemic and hemorrhagic stroke differ [10]. Additional risk factors for ischemic stroke include diabetic kidney disease, poor glycemic control, and insulin resistance, while for hemorrhagic stroke the presence of diabetic kidney disease and diabetic retinopathy, and lower BMI increases the risk [10, 11].

In addition to modifiable risk factors, genetic risk factors are also important for the development of stroke. A large genome-wide association study identified different loci for ischemic and hemorrhagic stroke. [12] To evaluate the genetic risk in routine clinical practice, a simple tool is to assess the family history and parental risk factors. Studies on parental risk factors show parental stroke to be associated with increased risk of stroke in their offspring [13, 14]. It has also been shown that hypertension and stroke occur concurrently in families [15]. In type 1 diabetes the impact of parental risk factors on the risk of stroke has not been studied. Our aim was, therefore, to explore a potential familial predisposition to stroke and specifically how hypertension, diabetes, stroke, and/or myocardial infarction in the parents of individuals with type 1 diabetes affect their risk of stroke.

## Methods

All individuals are part of the Finnish Diabetic Nephropathy (FinnDiane) Study, which is a multicenter study founded in 1997, including 77 study centers (Additional file 1). The study aims to uncover genetic and environmental risk factors for micro- and macro-vascular complications in type 1 diabetes. This study is an observational follow-up study, previously described in detail [16]. Of the 4,467 individuals with type 1 diabetes that have participated in the FinnDiane study before 1.1.2013, we excluded 350 with incomplete parental history, 96 with stroke prior to baseline, and 10 with traumatic or uncertain stroke, resulting in 4011 eligible individuals for the study. Type 1 diabetes was defined as age at onset below 40 years and initiation of insulin therapy within one year from diagnosis.

The study protocol has been approved by the Ethics Committee of the Helsinki and Uusimaa Hospital District. Each individual gave their written informed consent prior to their inclusion in the study. The study was conducted in accordance with the Declaration of Helsinki.

Data were collected at regular visits to the attending physician. Information regarding medical history and medication was registered. Anthropometrics were measured at the visit (body weight, height, and blood pressure). Blood samples were drawn and analyzed for HbA_1c_, lipids and lipoproteins, as well as serum creatinine. The glomerular filtration rate (eGFR) was estimated with the Chronic Kidney Disease Epidemiology Collaboration (CKD-EPI) equation [17]. Diabetic kidney disease was defined as history of severely increased albuminuria in two out of three urine collections (≥ 200 µg/min or ≥ 300 mg/24 h), history of renal transplantation, or being on dialysis. Cardiovascular disease was defined as history of myocardial infarction, coronary revascularization, and/or lower limb amputation or revascularization. History of smoking was defined as previous or current smoking. The participating individuals filled out questionnaires concerning their medical history, smoking habits, and family history. In this study, we used the information available from the questionnaires regarding the medical history of the parents. The questionnaires on parental history included information on year of birth and death, history of antihypertensive treatment, myocardial infarction, stroke, and diabetes. In case a parent had diabetes, the age at diagnosis (< 30, 30–60, or > 60 years) and type of treatment (diet, oral hypoglycemic agents, or insulin) was documented. Based on this the condition could be defined as type 1 diabetes (age at onset < 30 years and insulin treatment), type 2 diabetes (age at onset > 60 years or treatment with oral hypoglycemic agents or diet), or unclassifiable diabetes (age between 30 and 60 at onset or no information on age at onset or type of medication).

*Follow-up data.* Individuals who suffered a stroke were identified from death certificates retrieved from Statistics Finland and from the Finnish Care Register for Health Care based on the ICD-10 (codes I60-I64). Medical records, computed tomography, and magnetic resonance images were then ordered for these identified individuals from the hospital where they had been treated. All strokes were verified by a stroke neurologist (J.P.) and classified as either ischemic or hemorrhagic according to a protocol previously described in detail [2, 11]. Follow-up data were available until 31 December 2012.

*Statistical analyses*. We tested all continuous variables for normal distribution. For parametric continuous variables, we used *t* tests to compare means, and for non-parametric ones, we used Mann–Whitney U-tests. The results are presented as mean with standard deviation or as median with interquartile intervals, respectively. We used the ***χ***^**2**^-test to test differences in categorical variables between groups.

Cox regression analyses were conducted to assess the independent role of parental risk factors for the first ever stroke during follow-up, as well as for ischemic or hemorrhagic stroke, respectively. Variables were chosen based on the significance in univariable analysis, and the final models included variables independently associated with the outcome. The final model for the Cox regression analysis for any stroke included sex, age, systolic blood pressure, HbA_1c_, retinal photocoagulation, and diabetic kidney disease. The Cox regression analysis for ischemic stroke included the same variables as for any stroke, with the addition of smoking, whereas the analyses for hemorrhagic stroke included sex, age, BMI, retinal photocoagulation, and diabetic kidney disease. Each parental risk factor was then added to the model separately. The results are presented as hazard ratio with 95% confidence interval. *p* < 0.05 was considered statistically significant. Statistical analyses were performed with SPSS Statistics 24.0 software (IBM Corporation, Armonk, NY, USA) and R version 4.0.0.

## Results

During a median follow-up of 12.4 (10.9–14.2) years, 188 (4.6%) of the 4,011 study participants were diagnosed with their first ever stroke; 134 (71.3%) were ischemic and 54 (28.7%) were hemorrhagic. The characteristics of the individuals and their parents at baseline, according to the presence or the absence of stroke during follow-up, are shown in Table 1. Individuals with stroke were older, were more often men, and had a longer diabetes duration. Higher blood pressure, unfavorable cholesterol concentrations, higher HbA_1c_, lower eGFR, and a history of diabetic kidney disease, retinal photocoagulation, and cardiovascular disease, as well as smoking were more common among the individuals with stroke.

**Table 1:** Characteristics of the participants at baseline according to presence or absence of stroke during follow-up

	No stroke	Any stroke	Ischemic stroke	Hemorrhagic stroke
	*n* = 3,823	*n* = 188	*n* = 134	*n* = 54
Men (%)	51	62*	62*	61
Age, years	37.2 ± 11.8	45.8 ± 9.5^†^	46.7 ± 9.6^†^	43.6 ± 8.9^†^
Age at onset, years	14.0 (9.2–22.3)	13.0 (7.9–21.7) ^†^	13.2 (8.0–23.4)	12.8 (7.5–17.1)
Duration of diabetes, years	20.4 (11.3–30.0)	31.0 (24.9–36.8) ^†^	31.8 (25.1–37.5) ^†^	29.1 (24.3–36.3) ^†^
BMI, kg/m^2^	25.0 ± 3.6	25.0 ± 3.8	25.4 ± 3.9	24.0 ± 3.4
Systolic blood pressure, mmHg	133 ± 18	148 ± 23^†^	150 ± 23^†^	145 ± 23^†^
Diastolic blood pressure, mmHg	79 ± 10	82 ± 11^†^	82 ± 11*	83 ± 12*
Total cholesterol, mmol/l	4.90 ± 0.96	5.39 ± 1.10^†^	5.37 ± 1.05^†^	5.41 ± 1.22*
LDL cholesterol, mmol/l	2.99 ± 0.87	3.39 ± 1.00^†^	3.41 ± 0.96^†^	3.34 ± 1.10*
HDL cholesterol, mmol/l	1.34 ± 0.39	1.29 ± 0.41	1.27 ± 0.41*	1.33 ± 0.42
Triglycerides, mmol/l	1.01 (0.77–1.44)	1.30 (0.94–1.92) ^†^	1.27 (0.94–1.90) ^†^	1.32 (0.96–1.96) ^†^
HbA_1c_, % (mmol/mol)	8.37 ± 1.48 (67.9 ± 16.1)	8.88 ± 1.29 (73.5 ± 14.1)^†^	8.88 ± 1.23 (73.5 ± 13.4)^†^	8.87 ± 1.46 (73.5 ± 16.0)*
eGFR, ml/min/1.73 m^2^	100 (83–114)	68 (38–97) ^†^	70 (44–98) ^†^	64 (34–97) ^†^
Diabetic kidney disease, % (*n*)	20.3 (722)	60.2 (112) ^†^	59.8 (79) ^†^	61.1 (33) ^†^
Retinal photocoagulation, % (*n*)	31.4 (1,192)	74.7 (139) ^†^	75.2 (100) ^†^	73.6 (39) ^†^
Cardiovascular disease, % (*n*)	6.6 (252)	20.7 (39) ^†^	24.6 (33) ^†^	11.1 (6)
History of smoking, % (*n*)	45.2 (1,702)	61.0 (114) ^†^	64.2 (86) ^†^	52.8 (28)
*Maternal characteristics*				
Age, years	61.0 (53.0–70.0)	71.0 (65.0–76.0) ^†^	72.0 (65.0–76.0) ^†^	69.0 (62.8–75.5) ^†^
Age at death, years	70.0 (59.0–78.0)	73.0 (67.0–80.0)*	72.0 (64.5–80.8)	76.0 (69.0–77.0)
Mother alive, % (*n*)	81.6 (3,118)	64.4 (121) ^†^	62.7 (84) ^†^	68.5 (37)*
Hypertension, % (*n*)	33.4 (1,162)	42.1 (69)*	42.2 (49)*	41.7 (20)
Myocardial infarction, % (*n*)	7.5 (254)	17.3 (28) ^†^	19.0 (22) ^†^	13.0 (6)
Stroke, % (*n*)	4.2 (142)	11.9 (19) ^†^	9.6 (11)*	17.4 (8) ^†^
Diabetes, % (*n*)	13.0 (461)	19.2 (33)*	19.8 (24)*	17.6 (9)
*Paternal characteristics*				
Age, years	61.0 (54.0–69.0)	72.0 (61.5–75.0) ^†^	74.0 (61.5–76.0) ^†^	70.5 (61.0–73.8)^**†**^
Age at death, years	62.9 ± 14.2	65.5 ± 12.6	65.4 ± 12.4	66.0 ± 13.6
Father alive, % (*n*)	65.1 (2,453)	38.0 (71) ^†^	34.6 (46) ^†^	46.3 (25)*
Hypertension, % (*n*)	29.0 (921)	32.9 (48)	33.7 (35)	31.0 (13)
Myocardial infarction, % (*n*)	20.0 (644)	29.6 (45)*	28.7 (31)*	31.8 (14)
Stroke, % (*n*)	5.8 (183)	7.8 (12)	8.3 (9)	6.8 (3)
Diabetes, % (*n*)	14.3 (476)	17.7 (29)	19.0 (22)	14.6 (7)

Data are mean ± SD, median (interquartile range), or percentage (%). *p*-values indicate comparison with no stroke. *eGFR* , estimated glomerular filtration rate.

*p < 0.05, ^†^*p* < 0.001

**Figure 1:**
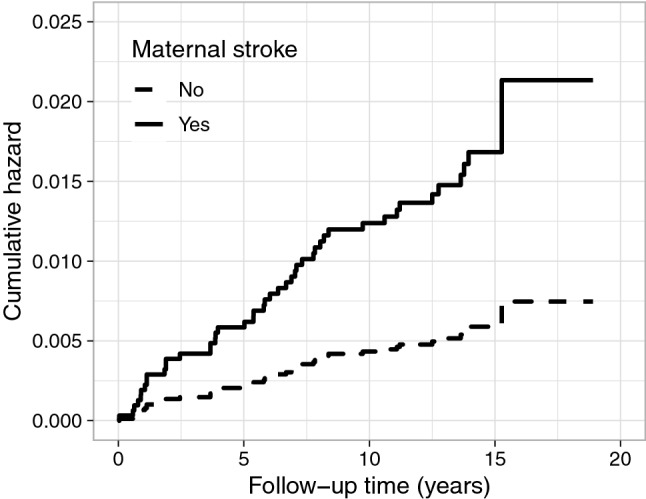
Cumulative hazard for hemorrhagic stroke according to maternal stroke. Maternal stroke associated with hemorrhagic stroke, hazard ratio 2.86 (95% CI 1.27–6.44), *p* = 0.011, adjusted for sex, age, BMI, retinal photocoagulation, and diabetic kidney disease

Since the individuals with stroke were older, also their parents were older at baseline, resulting in a higher prevalence of parental risk factors. The prevalence of stroke, myocardial infarction, hypertension, and diabetes was higher for the mothers of individuals with stroke, especially in the any stroke and ischemic stroke groups. (Table 1) After adjustment for age, only maternal stroke was associated with any stroke (*p* = 0.040) and hemorrhagic stroke (*p* = 0.010). Further, in Cox regression analysis, only maternal stroke was associated with increased risk of hemorrhagic stroke in individuals with type 1 diabetes, hazard ratio 2.86 (95% CI 1.27–6.44, *p* = 0.011) (Fig. 1, Table 2).

**Table 2:** Associations between parental risk factors and stroke during follow-up

	Any stroke (*n* = 188)	*p*-value	Ischemic stroke(*n* = 134)	*p*-value	Hemorrhagic stroke(*n* = 54)	*p*-value
*Maternal risk factors*						
Hypertension	1.01 (0.73–1.40)	0.937	0.98 (0.67–1.44)	0.917	1.14 (0.63–2.05)	0.671
Myocardial infarction	1.35 (0.87–2.07)	0.179	1.46 (0.90–2.37)	0.130	1.18 (0.49–2.83)	0.717
Stroke	1.49 (0.90–2.46)	0.119	1.13 (0.59–2.15)	0.717	2.86 (1.27–6.44)	0.011
Diabetes	1.07 (0.73–1.59)	0.720	0.96 (0.61–1.52)	0.859	1.10 (0.53–2.30)	0.799
*Paternal risk factors*						
Hypertension	1.05 (0.73–1.50)	0.810	1.13 (0.74–1.72)	0.567	0.99 (0.50–1.95)	0.977
Myocardial infarction	1.16 (0.81–1.67)	0.417	1.16 (0.76–1.77)	0.501	1.44 (0.74–2.82)	0.286
Stroke	0.98 (0.54–1.77)	0.938	0.99 (0.50–1.98)	0.985	0.83 (0.26–2.72)	0.762
Diabetes	1.26 (0.83–1.89)	0.276	1.42 (0.89–2.27)	0.143	0.93 (0.39–2.20)	0.865
*Parental risk factors*						
Hypertension	0.97 (0.69–1.36)	0.846	0.90 (0.60–1.35)	0.610	1.21 (0.64–2.31)	0.560
Myocardial infarction	1.28 (0.91–1.80)	0.160	1.32 (0.88–1.98)	0.174	1.32 (0.70–2.51)	0.393
Stroke	1.23 (0.81–1.88)	0.326	1.03 (0.61–1.73)	0.915	1.88 (0.92–3.86)	0.085
Diabetes	1.14 (0.82–1.59)	0.431	1.22 (0.82–1.80)	0.325	0.92 (0.48–1.75)	0.791

Adjusted Cox regression analyses for association between parental risk factors at baseline and any stroke, ischemic stroke, and hemorrhagic stroke, respectively, during a median of 12.4 years follow-up. Data are hazard ratios with 95% confidence intervals. For any stroke, the analyses were adjusted for sex, age, systolic blood pressure, HbA_1c_, retinal photocoagulation, and diabetic kidney disease. For ischemic stroke, the analyses were adjusted for the same variables as for any stroke, as well as smoking. For hemorrhagic stroke, the analyses were adjusted for sex, age, BMI, retinal photocoagulation, and diabetic kidney disease. All parental risk factors were analyzed separately

Of the paternal risk factors, only the prevalence of myocardial infarction was higher for the fathers of individuals with any stroke and ischemic stroke. Noteworthy, at baseline, fewer fathers than mothers were alive, and even fewer of the fathers to those that had suffered a stroke were alive. (Table 1). In Cox regression analyses, no significant associations between parental hypertension, myocardial infarction, stroke, and diabetes and the risk of stroke or stroke subtypes appeared (Table 2).

## Discussion

In this large observational follow-up study of individuals with type 1 diabetes, we showed that maternal stroke is associated with increased risk of stroke, especially hemorrhagic stroke. None of the other parental risk factors studied were associated with increased risk of stroke or the stroke subtypes ischemic or hemorrhagic. Our results indicate that familial predisposition to stroke plays a lesser role in type 1 diabetes and that diabetes-related factors, in contrast, play a larger role.

This is the first study, to our knowledge, to assess parental risk factors for risk of stroke in type 1 diabetes. We showed that maternal stroke increases the risk of hemorrhagic stroke by nearly threefold after adjustment for confounders. Similarly, in people without diabetes, parental stroke is associated with a threefold increased risk of stroke in the offspring [13], and familial predisposition to stroke is stronger for hemorrhagic stroke than for ischemic [18]. For the different subtypes of stroke, the familial predisposition differs, as shown by a large Swedish population-based study, where the type of stroke in siblings had different impact on the stroke subtype in the index case. Hemorrhagic stroke in siblings increased the risk of hemorrhagic stroke, while ischemic stroke in siblings increased the risk of ischemic stroke, but not vice versa. [19] In our study, we did unfortunately not have data on the subtype of stroke in the parents or data on stroke in siblings.

In line with our results, maternal history is more prevalent than paternal history of stroke in individuals with stroke, especially in those with ischemic stroke and in particular women with stroke [20]. In our study, we observed similar findings, but for those with hemorrhagic stroke. It was not possible to assess the parental risk factors separately for men and women with type 1 diabetes, due to lack of statistical power. However, in individuals with type 1 diabetes, we previously observed that men have a higher risk of stroke than women, but this difference is explained by the higher risk of diabetic kidney disease in men [10], and therefore all analyses were adjusted for both sex and diabetic kidney disease in the present study. In contrast to studies in the general population [15, 21], we did not observe an association between parental hypertension and risk of stroke. It is possible that the familial predisposition to stroke is of lesser importance in individuals with type 1 diabetes. This risk of stroke is foremost driven by diabetes-related factors, such as diabetic kidney disease, which has been shown to have a strong impact on the risk of stroke in the individuals in whom stroke occurs at a relatively young age [2]. In this case, a less prominent familial predisposition could be masked by the presence of diabetic kidney disease. In addition, parental hypertension, and maternal hypertension, in particular, has previously been shown to be associated with diabetic kidney disease in type 1 diabetes [22], which might further complicate the interpretation of the data. Due to the low number of stroke events in the individuals without diabetic kidney disease, we were unable to assess the impact of parental risk factors in these individuals separately.

We also assessed the impact of parental history of myocardial infarction and parental diabetes on the risk of stroke and found neither of these to increase the risk of any stroke or stroke subtypes. Parental history of myocardial infarction could indicate a history of atherosclerosis. In type 1 diabetes, the stroke etiology differs from the general population in that a larger proportion of the stroke events are caused by small-vessel disease, whereas large-vessel atherosclerosis is less common [23]. This may explain the lack of association between parental myocardial infarction and risk of stroke in the present study. On the other hand, no such association has been demonstrated for individuals without diabetes either [14].

The strength of this study is the large well-characterized cohort of individuals with type 1 diabetes, with all strokes verified from the medical files by an experienced stroke neurologist. Although the cohort is large, the number of events did not allow extensive sub-analyses, for example, according to sex or absence of diabetic kidney disease. The information about the parents was obtained from the individuals with type 1 diabetes by a questionnaire. Ideally, we would have obtained the information about the parents’ medical history by examining them directly, but this was not feasible in the large nationwide FinnDiane cohort. However, the questionnaire enabled us to get information also on those parents that had died. Compared to the mothers, more fathers had died, and they had died at a younger age, which could impact the results regarding the lack of association between paternal risk factors and stroke. Another limitation is that we have only studied the risk factors in the parents, although the medical history of siblings could have an even larger impact on the stroke risk [24].

## Conclusions

In conclusion, we observed that a history of maternal stroke is a significant risk factor for hemorrhagic stroke in individuals with type 1 diabetes. Other parental risk factors seem to have limited impact on the risk, and there is a need for further studies to validate these findings.

## Supplementary Information

Supplementary material 1 (PDF 158 kb)

## Data Availability

The datasets generated and/or analyzed during the current study are not publicly available due to the local legislation and the written consents of the FinnDiane study participants, which do not allow sharing individual-level phenotype data. The data that support the findings are available from the corresponding author upon reasonable request.

## Abbreviations

CI: Confidence interval
eGFR: Estimated glomerular filtration rate
FinnDiane: The Finnish Diabetic Nephropathy Study
